# EFEN-YOLOv8: Surface defect detection network based on spatial feature capture and multi-level weighted attention

**DOI:** 10.1371/journal.pone.0339617

**Published:** 2026-01-02

**Authors:** Meishun Wu, Jinmin Peng, Xinyi Yu, Heng Xu, Haotian Sun

**Affiliations:** 1 School of Mechanical and Automotive Engineering, Fujian University of Technology, Fuzhou, China; 2 Fujian Key Laboratory of Intelligent Processing Technology and Equipment, Fujian University of Technology, Fuzhou, China; Hohai University, CHINA

## Abstract

Surface defects in industrial environments severely the impact product aesthetics, quality, and operational efficiency. Although deep learning approaches show promise, current architectures often demonstrate inadequate feature extraction in industrial settings. We introduce EFEN-YOLOv8, a novel defect detection framework that prioritizes efficient feature extraction to enhance detection accuracy. Our approach incorporates a β-FEIoU loss function that concurrently tackles defect-background discrimination and positive-negative sample imbalance. The Shallow Attention Convolution (SAConv) module strengthens feature localization in early network layers, while Large Separable Kernel Attention (LSKA) expands receptive fields and augments processing efficiency. Additionally, our Weighted Atrous Spatial Pyramid Pooling (WASPP) feature fusion module facilitates multi-scale integration, enabling richer abstract information capture and improved model representation. Comprehensive experimental validation, including statistical significance testing across diverse data splits, confirms superior performance over existing methods. Our framework achieves 7.4% mAP improvement on NEU-DET and 3.3% enhancement on GC10-DET compared to baseline models, maintaining consistent performance across both 8:2 and 9:1 train-test configurations. These findings validate the method’s robust generalization capacity and establish its effectiveness for industrial surface defect detection applications. Code and datasets are available at: https://github.com/01WineCool/YOLO.

## Introduction

Materials across automotive, aerospace, construction, and manufacturing sectors exhibit specialized properties tailored to demanding operational requirements. Nevertheless, environmental exposure and processing conditions induce surface defects including scratches, pitting, corrosion, and delamination, which degrade both aesthetic appeal and structural integrity while diminishing service longevity. Conventional detection approaches rely predominantly on manual visual inspection, introducing human error, fatigue-related inconsistencies, elevated operational costs, and reduced throughput—limitations that render such methods insufficient for contemporary industrial applications. Alternative methodologies encompass laser scanning, magnetic flux leakage testing, eddy current evaluation, and ultrasonic inspection; however, these techniques demand rigorous technical specifications, impose substantial capital expenditures, and exhibit susceptibility to environmental interference, thereby constraining their widespread deployment in production environments [1].

Advances in automation and computer vision have catalyzed innovative surface defect detection paradigms. Initial methodologies employed manual feature extraction coupled with image preprocessing prior to classification. Ashour et al. [2] utilized gray-level co-occurrence matrices (GLCM) to extract multi-directional texture features from steel strip imagery for defect identification. Similarly, Carvalho et al. [3] developed pixel clustering techniques through specialized feature extraction, while Schneider et al. [4] captured signal characteristics across temporal, spectral, and distributional domains, incorporating feature selection mechanisms for enhanced recognition accuracy. Despite achieving adequate performance within specific operational contexts, these approaches exhibit domain-specific feature extraction constraints, fundamentally limiting their generalizability and posing substantial scalability challenges.

Deep learning has revolutionized defect detection through its superior representational capabilities, with optimized object detection algorithms further advancing detection efficiency. Two-stage methodologies, exemplified by the R-CNN family [5,6], achieve exceptional accuracy through region proposal mechanisms, while single-stage approaches including YOLO variants [7,8] and SSD architectures [9] excel in real-time scenarios due to their computational efficiency. Recent advances demonstrate continued innovation: Sun et al. [10] enhanced R-CNN performance by incorporating attention-guided feature encoding and multi-level grid-based ROI fusion modules. Liu et al. [11] strengthened feature extraction through FocalNextBlock integration within backbone architectures. The transformer-based DETR [12] pioneered end-to-end single-stage detection, while RT-DETR [13] addressed computational overhead concerns despite maintaining substantial parameter requirements. Wei et al. [14] proposed a vision transformer combining receptive-field attention convolution (RFAConv) with context broadcasting median (CBM) modules, achieving significant improvements in metallic surface defect detection.

Surface defects present multifaceted challenges characterized by irregular geometries, variable dimensions, inconsistent aspect ratios, and heterogeneous illumination patterns. Such variability undermines detection algorithm effectiveness, while complex backgrounds impose additional computational burdens on parameter-intensive networks. Object detection frameworks encounter particular difficulties with multi-scale generalization, necessitating extensive downsampling operations that compromise small target detection capabilities. In scenarios dominated by intricate backgrounds and minute defective regions, feature extraction becomes exceptionally demanding. Single-stage architectures offer optimal solutions for time-critical applications, achieving favorable speed-accuracy trade-offs through unified detection pipelines that enhance practical deployment viability. However, significant optimization opportunities persist across diverse industrial contexts. Given that model performance varies substantially across application domains, strategic detector selection remains paramount for maximizing inspection efficiency. This work presents EFEN-YOLOv8, an enhanced framework built upon YOLOv8 foundations, specifically designed for robust defect detection across complex industrial environments. The principal contributions include:

- We introduce a novel Shallow Attention Convolution (SAConv) module, which employs fine-grained attention mechanisms to significantly enhance spatial feature localization and positional encoding. Crucially, this module facilitates precise feature extraction within early network layers. Complementing SAConv, our innovative Weighted Atrous Spatial Pyramid Pooling (WASPP) module ensures robust multi-scale feature integration and comprehensive contextual information preservation.
- To address both the challenging foreground-background discrimination and the pervasive class imbalance inherent in defect detection datasets, we propose a novel β-FEIoU loss function. This innovative formulation concurrently optimizes bounding box regression while effectively mitigating the significant positive-negative sample disparity frequently encountered in real-world industrial inspection scenarios.
- Our proposed framework achieves exceptional performance, establishing new competitive baselines on the challenging NEU-DET and GC10-DET benchmarks. These results unequivocally advance the state-of-the-art in surface defect detection.

## Related work

Deep learning-based object detection has emerged as the dominant paradigm for surface defect detection, driven by advances in novel convolution architectures, attention mechanisms, loss function optimization, and multi-scale feature fusion strategies.

**Convolution methods.** Convolutional operations extract hierarchical feature representations while enabling spatial dimension manipulation through learnable filters. Unlike fully connected architectures, convolutional layers achieve parameter efficiency, motivating extensive research into specialized convolution variants. Dent et al. [15] developed Spatial Depth Convolution (SPD-Conv) blocks that preserve complete channel information through feature map-specific operations, demonstrating enhanced performance on low-resolution imagery and small object detection. Wang et al. [16] combined self-attention with graph convolution while employing lightweight Depthwise Convolution (DWconv) modules, achieving both computational acceleration and improved recognition of challenging defect patterns. Zhong et al. [17] introduced DualConv, which processes identical input channels through parallel 3×3 and 1×1 kernels, optimizing feature extraction and yielding substantial accuracy improvements in YOLOv3 implementations. Shahaf et al. [18] leveraged Wavelet Transform (WT) for multi-frequency analysis, capturing rich low-frequency components and global receptive fields through convolution-based spatial mixing, thereby enhancing CNN robustness against geometric variations and structural damage. Chen et al. [19] proposed Partial Convolution (PConv), applying standard convolution to selected input channels while maintaining others unchanged, significantly reducing computational overhead and memory consumption. Each technique offers distinct computational and representational advantages, necessitating careful selection based on specific application requirements and performance constraints.

**Attention mechanisms.** While convolution excels at local feature extraction, attention mechanisms provide global contextual modeling capabilities that enhance long-range dependency capture and enable selective focus on salient input regions. Li et al. [20] integrated dual channel-spatial attention to strengthen feature fusion within neural architectures. Tang et al. [21] developed joint attention frameworks that suppress background interference while emphasizing defect characteristics, thereby improving network analytical capabilities. However, enhanced attention modules may introduce computational overhead and convergence challenges. Guo et al. [22] proposed large kernel attention that unifies convolutional and self-attention advantages, incorporating local structural modeling, long-range dependency capture, and adaptive receptive field adjustment. Kang et al. [23] addressed computational efficiency in global context extraction through Channel Reduction Attention (CRA), which compresses query and key dimensions to unity, substantially reducing self-attention computational complexity. Wang et al. [24] focused on multi-head attention for detail preservation and inference acceleration, employing jump-sensitive feature fusion modules that maintain texture extraction fidelity while enhancing detection performance. Despite these advances, many attention mechanisms remain constrained by their limited receptive fields, hampering fine-grained feature capture essential for detecting minute or subtle surface anomalies.

**Loss functions.** Loss function optimization represents a critical pathway for enhancing model stability and convergence efficiency, directly influencing both training dynamics and regression precision. Hu et al. [25] synthesized mean squared error, cross-entropy, and CIoU components into a unified loss formulation that constrains classification performance while improving model stability and accuracy. Liao et al. [26] designed an angular-based loss function that considers geometric relationships between predicted and ground truth centroids, balancing component contributions through L1 and Alpha-IoU integration to achieve superior detection accuracy and accelerated convergence. Li et al. [27] directly incorporated localization quality into classification loss through category-specific weighting schemes, enabling detection models to prioritize challenging samples and address classification-localization inconsistencies inherent in traditional object detection paradigms. Luo et al. [28] introduced UNI-IoU loss for bounding box regression, optimizing accuracy through dynamic attention mechanisms that adapt to prediction quality variations. While existing loss functions primarily minimize spatial discrepancies between predictions and the ground truth, they seldom account for inter-class relationships among defect categories, representing a significant opportunity for improvement.

**Multi-scale fusion.** Despite these advances, small defect detection remains challenging due to insufficient multi-scale feature integration and extraction capabilities. Recent investigations [1,26,29,30] have incorporated high-resolution feature maps containing rich small object information into fusion networks, employing sophisticated bidirectional feature pyramid networks (Bi-FPN) to enhance small target detection accuracy. Li et al. [31] combined top-down upsampling with bottom-up downsampling pathways to emphasize both positional and semantic information within multi-scale fusion architectures. Zhang et al. [32] integrated Atrous Spatial Pyramid Pooling (ASPP) modules into YOLOv5, substantially improving small target detection performance on limited datasets through diverse semantic information capture. Li et al. [33] incorporated simplified Spatial Pyramid Pooling-Fast (SimSPPF) structures into backbone networks, enabling feature extraction across four distinct scales. Yang et al. [34] proposed FocalModulation for feature enhancement, employing attention mechanisms to concentrate on salient image regions and improve regional recognition capabilities. Compared to SPPF, FocalModulation processes variable-sized inputs while achieving superior object identification and localization precision.

While these methodologies demonstrate effectiveness in specific contexts, they exhibit fundamental limitations in complex scenarios. Shallow network layers typically contain noisy, low-level features, whereas deeper layers often fail to preserve fine-grained spatial details essential for precise localization. This hierarchical information disparity impedes effective attention allocation to critical regions. Although ASPP and similar architectures successfully integrate multi-level information, they inadequately prioritize discriminative features, constraining model enhancement potential and limiting breakthrough performance. To address these challenges, detection frameworks must exhibit superior global modeling capabilities, effectively discriminate between heterogeneous features, and assess their relative importance for decision-making. Through enhanced shallow feature attention and comprehensive local-global information integration, our proposed architecture achieves more effective feature discrimination and superior detection performance.

## Methods

### YOLOv8 algorithm and EFEN-YOLOv8 architecture

**Fig 1:**
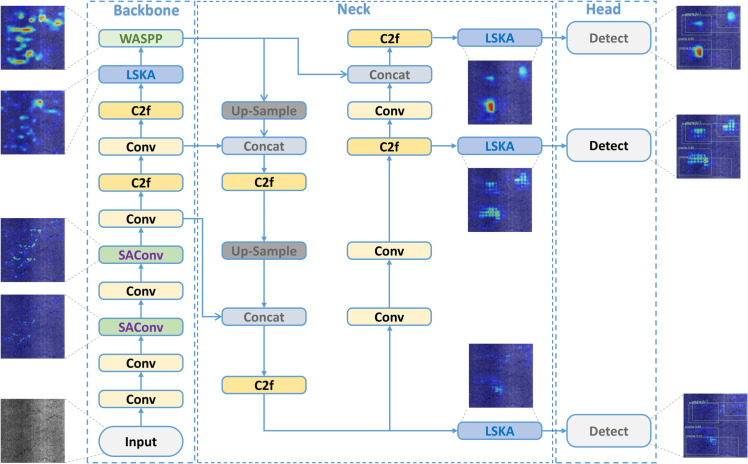
EFEN-YOLOv8 architecture. Thermal maps demonstrate the effectiveness of each improvement component. The enhanced model successfully captures defect information at shallow layers while maintaining focus on defect features through multi-scale fusion and attention mechanisms.

The YOLOv8 framework consists of four principal components: backbone feature extraction network, neck feature fusion network, detection head, and loss function incorporating both classification and localization objectives. Our EFEN-YOLOv8 architecture, depicted in Fig 1, introduces several key modifications to address defect detection challenges. We replace the initial two C2f modules in the backbone with SAConv modules that provide expanded receptive fields and weighted attention mechanisms. LSKA attention is strategically integrated before fusion layers and detection heads to strengthen defect feature identification capabilities. Our novel WASPP module emphasizes feature importance while integrating diverse semantic information across multiple scales. Finally, we propose the β-FEIoU loss function to enhance defect discrimination and mitigate positive-negative sample imbalance inherent in defect detection datasets.

### Shallow Attention Convolution (SAConv)

Hierarchical feature learning in deep convolutional networks reveals distinct representational characteristics across network depths. Shallow layers, positioned proximal to input data, preserve fine-grained spatial information including texture patterns, edge structures, and low-level visual primitives essential for precise localization. Conversely, deeper layers progressively abstract semantic concepts through nonlinear transformations, enabling high-level reasoning and complex pattern recognition. The quality of early-stage feature learning fundamentally influences the subsequent network performance, as initial representations form the foundation for all downstream processing. However, standard convolutional operations face inherent limitations in establishing long-range spatial dependencies due to constrained receptive fields, particularly affecting early layers where fine-grained spatial relationships are crucial.

**Fig 2:**
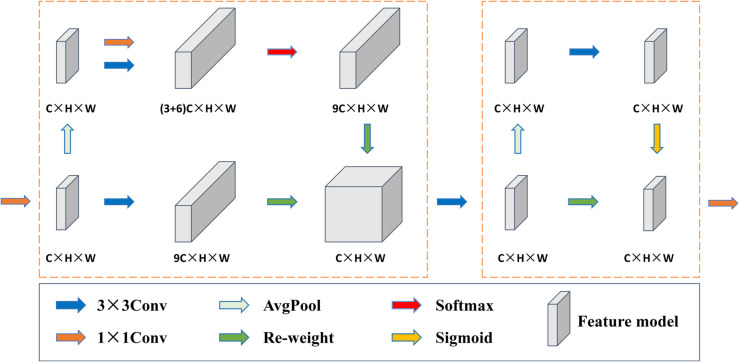
SAConv module architecture and computational flow. The module employs multi-scale kernel operations followed by adaptive pooling and attention mechanisms for enhanced shallow feature extraction.

For surface defect detection, shallow layer information proves indispensable for identifying minute anomalies and subtle textural variations. Traditional CNNs often inadequately preserve and refine these critical shallow features due to limited spatial connectivity and insufficient attention mechanisms. To address these limitations and enhance shallow-layer defect sensitivity, we introduce the Shallow Attention Convolution (SAConv) module, specifically engineered to strengthen fine-grained feature representation while maintaining computational efficiency. The SAConv operational mechanism is illustrated in Fig 2.

SAConv operates through a dual-stage architecture optimized for shallow feature enhancement. The initial stage captures multi-scale spatial dependencies through heterogeneous convolutional kernels of varying dimensions. Small kernels preserve fine-grained detail resolution essential for detecting subtle defects, while larger kernels extend spatial receptive fields to capture broader contextual information. This multi-kernel interaction enables comprehensive spatial relationship modeling across diverse scales, facilitating robust feature learning that spans both local and semi-global spatial contexts. The subsequent stage employs adaptive pooling operations coupled with attention mechanisms to refine feature localization and importance weighting. This pooling strategy ensures effective aggregation of scattered defect information while attention mechanisms amplify discriminative features critical for accurate detection. Through this integrated approach, SAConv maintains rich spatial detail at shallow network depths while enhancing sensitivity to subtle anomalies, ultimately improving detection performance with minimal computational overhead.

### β-FEIoU loss function

Inter-class spatial relationships in surface defect detection significantly impact classification performance, particularly when visually similar defect categories exhibit overlapping characteristics. Certain defect types demonstrate strong structural similarities during acquisition, such as comparable grayscale distributions between oil stains and water reflections on metallic surfaces. These visual ambiguities compromise the network discrimination capabilities, resulting in suboptimal feature learning and classification errors. Conventional loss functions assume simplified feature distributions and inadequately account for nuanced inter-category similarities, often treating distinct yet visually comparable defects as indistinguishable. This limitation manifests as discrimination deficiencies when structural differences between classes remain subtle yet critical for accurate categorization.

We propose the β-FEIoU (Feature-Enhanced Intersection over Union) loss function to enhance model sensitivity to fine-grained class distinctions. The β-FEIoU formulation prioritizes spatial and structural variations between similar categories, enabling superior capture of discriminative features during training. As formulated in Eq (1), β-FEIoU incorporates an adaptive weighting factor β that dynamically modulates loss magnitude based on predicted-ground truth feature overlap. This mechanism emphasizes regions exhibiting high inter-class similarity, ultimately strengthening the model’s capacity to differentiate between visually comparable yet semantically distinct defects. Through enhanced spatial relationship modeling, β-FEIoU improves both classification accuracy and robustness across challenging detection scenarios.

$$L_{\beta \text{-FEIoU}}=\sum_{c=1}^{C}\beta (L_{\text{Focal}}+\lambda L_{\text{EIoU}}),$$ (1)

where *C* represents the total number of detection classes, β denotes the adaptive category weighting factor, and λ controls the relative contribution of the EIoU regression loss component.

**Fig 3:**
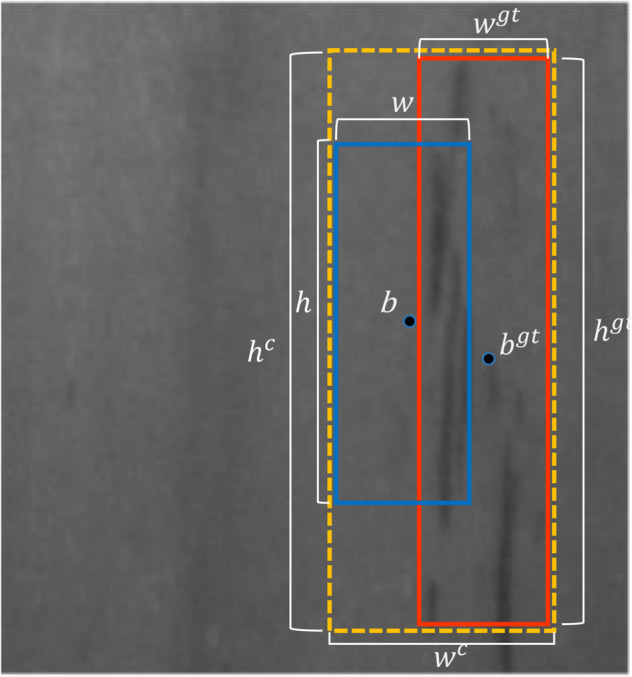
Geometric configuration for β-FEIoU computation. The predicted bounding box (blue), ground truth box (red), and minimum enclosing rectangle (yellow) define the spatial relationships used in loss calculation. Parameters *b*, *w*, and *h* represent box centers, widths, and heights respectively.

The EIoU loss function [35], detailed in Eq (2) and illustrated in Fig 3, enhances bounding box regression through comprehensive geometric penalty terms.

$$L_{\text{EIoU}}=1-\text{IoU}+\frac{d^{2}(b^{p},b^{gt})}{(w^{c}{)}^{2}+(h^{c}{)}^{2}}+\frac{d^{2}(w^{p},w^{gt})}{(w^{c}{)}^{2}}+\frac{d^{2}(h^{p},h^{gt})}{(h^{c}{)}^{2}},$$ (2)

where *w*^*c*^ and *h*^*c*^ represent the width and height of the minimum enclosing rectangle encompassing both predicted and ground truth boxes, and *d* denotes the Euclidean distance between specified points.

The Focal loss component addresses foreground-background imbalance prevalent in defect detection by dynamically reweighting training samples based on classification difficulty. This mechanism reduces the influence of easily classified samples while emphasizing hard negatives, effectively mitigating the positive-negative sample disparity characteristic of single-stage detection frameworks. The Focal loss formulation appears in Eq (3):

$$L_{\text{Focal}}=-\alpha_{t}(1-p_{t}{)}^{\gamma }\log(p_{t})$$ (3)

where

$$p_{t}=\left\{ \begin{array}{cc} p & \text{if }y=1\\ 1-p & \text{if }y=0 \end{array}\right.$$ (4)

where $\alpha_{t}$ represents the class-specific weighting factor, *p*_*t*_ denotes the predicted probability for the target class, and γ controls the focusing strength for hard example mining. When γ=0, the formulation reduces to standard cross-entropy loss.

### Large Separable Kernel Attention (LSKA)

**Fig 4:**
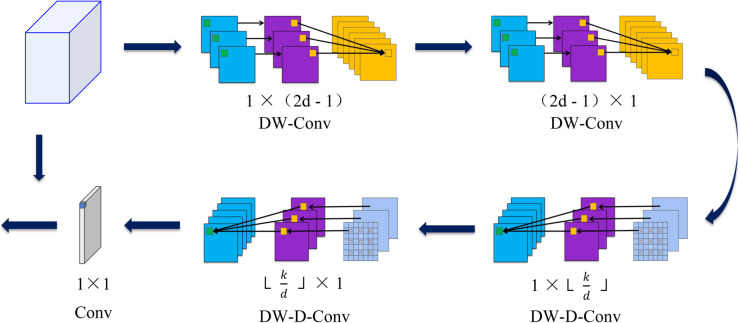
LSKA module architecture and computational flow. The module processes input features $F\in {\mathbb{R}}^{C\;\times \;H\;\times \;W}$ through cascaded depth-wise convolutions: standard DW-Conv followed by dilated DW-D-Conv operations. Results are concatenated with the original feature map after 1×1 convolution to produce the final attended output. Parameters: *C* denotes input channels, *H* and *W* represent spatial dimensions, *d* controls dilation rate, and *k* defines maximum receptive field extent.

Surface defects present multifaceted detection challenges characterized by irregular geometries, discontinuous boundaries, and complex edge artifacts, often compounded by substantial background noise and imaging interference. These conditions severely impede accurate defect localization within complex industrial environments. To enhance feature discrimination capabilities and improve defect characterization, we integrate the Large Separable Kernel Attention (LSKA) [36] mechanism preceding the final prediction layers. This strategic placement enables enhanced focus on critical defect characteristics while maintaining computational efficiency. As depicted in Fig 4, LSKA decomposes traditional 2D convolutional kernels into cascaded 1D separable operations, applied sequentially through depth-wise and dilated convolution pathways. This factorization strategy significantly reduces computational overhead and memory requirements while preserving large receptive field capabilities essential for capturing global spatial dependencies in complex defect patterns.

The LSKA mechanism operates through a multi-stage attention computation process:

$${\overset{―}{Z}}^{C}=\sum_{H,W}W_{(2d-1)\;\times \;1}^{C}*\left(\sum_{H,W}W_{1\;\times \;(2d-1)}^{C}*F^{C}\right)$$ (5)

$$Z^{C}=\sum_{H,W}W_{\left\lfloor \frac{k}{d}\right\rfloor \;\times \;1}^{C}*\left(\sum_{H,W}W_{1\;\times \;\left\lfloor \frac{k}{d}\right\rfloor }^{C}*{\overset{―}{Z}}^{C}\right)$$ (6)

$$A^{C}=W_{1\;\times \;1}*Z^{C}$$ (7)

$${\overset{―}{F}}^{C}=A^{C}⊗F^{C}$$ (8)

where * and ⊗ denote convolution and element-wise multiplication operations respectively. The intermediate feature ${\overset{―}{Z}}^{C}$ represents the depth-wise convolution output with kernel dimensions (2d−1)×(2d−1), capturing local spatial relationships while mitigating gridding artifacts inherent in dilated operations. The dilated convolution employs kernel size $\left\lfloor \frac{k}{d}\right\rfloor \;\times \;\left\lfloor \frac{k}{d}\right\rfloor$, where ⌊·⌋ represents the floor function. The attention map *A*${\;\;}^{\text{c}}$ results from 1×1 convolution for channel-wise feature recalibration. Through this hierarchical processing, dilated depth-wise convolution extracts global spatial context from local features ${\overset{―}{Z}}^{C}$, while maintaining computational efficiency through separable kernel decomposition.

Empirical analysis reveals that larger LSKA kernel sizes introduce computational overhead without proportional performance gains. Experimental validation demonstrates optimal detection accuracy at moderate kernel dimensions across both evaluation datasets, suggesting an effective balance between receptive field coverage and computational efficiency. A comprehensive kernel analysis can be found in the fourth section.

### Weighted Atrous Spatial Pyramid Pooling (WASPP)

Convolutional operations exhibit inherent spatial locality constraints, primarily capturing local patterns within limited receptive fields. While this locality enables effective fine-grained feature extraction, it fundamentally restricts global context modeling and high-level semantic understanding essential for comprehensive scene analysis. Multi-scale convolution strategies address this limitation by incorporating diverse receptive field configurations to capture hierarchical semantic information across spatial scales. However, features extracted from heterogeneous receptive fields exhibit varying relevance for specific detection tasks, with substantial portions potentially irrelevant to critical discriminative patterns.

**Fig 5:**
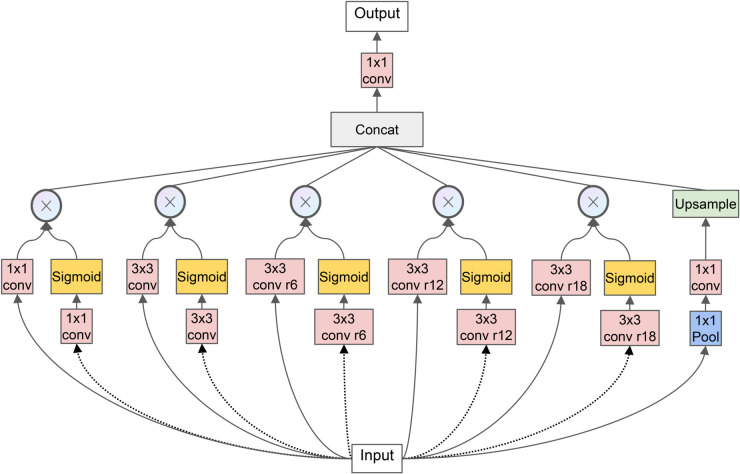
WASPP module architecture and multi-scale feature integration. The module employs parallel convolutional branches with varying receptive fields, followed by adaptive weighting mechanisms and feature concatenation. Each pathway contributes scale-specific information that is selectively emphasized through sigmoid-based attention before final fusion.

We propose the Weighted Atrous Spatial Pyramid Pooling (WASPP) module as an enhanced variant of traditional ASPP architectures, illustrated in Fig 5. WASPP integrates multiple parallel convolutional pathways employing standard kernels (1×1, 3×3) and atrous convolutions with systematic dilation rates (6, 12, 18) to capture multi-scale contextual information efficiently. This hierarchical design enables simultaneous extraction of local detail and global context while maintaining computational tractability for real-time applications.

To enhance discriminative feature selection, each convolutional branch within WASPP extracts features into dedicated channels, subsequently processed through sigmoid activation for adaptive importance weighting. This attention mechanism emphasizes salient features while suppressing irrelevant information, enabling selective focus on task-critical spatial patterns. The weighted features undergo concatenation with original convolutional outputs, facilitating comprehensive multi-scale representation that preserves both raw and refined feature information.

The module incorporates adaptive global average pooling followed by 1×1 convolution and bilinear upsampling to ensure consistent spatial dimensions across all feature pathways. Final feature integration occurs through concatenation and subsequent 1×1 convolution for dimensional consistency and feature refinement. This architecture effectively overcomes traditional convolution limitations in global context modeling by leveraging multi-scale kernel configurations and attention-guided feature selection. Through systematic integration of local and global information across diverse scales, WASPP enhances semantic understanding while maintaining computational efficiency, ultimately improving the model’s capacity to identify and localize complex defect patterns in challenging industrial environments.

## Experimental

To evaluate the effectiveness of our proposed framework, we conduct comprehensive experiments using the NEU-DET [37] dataset for initial module verification, followed by generalization validation on the GC10-DET [38] dataset.

### Datasets

#### NEU-DET dataset.

**Fig 6:**
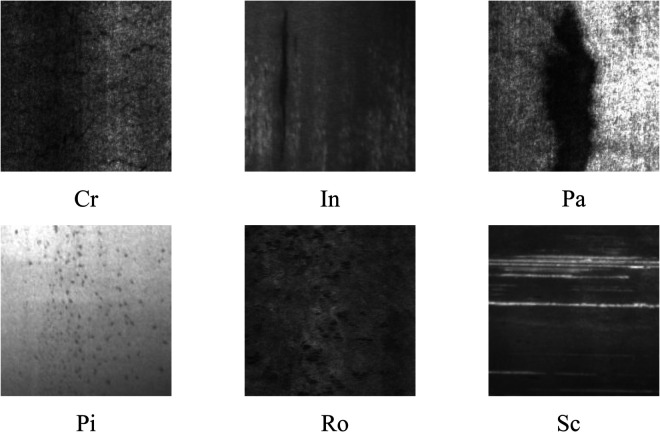
Representative defect categories in NEU-DET dataset. Each class exhibits distinct morphological characteristics and varying degrees of visual complexity, with irregular spatial distributions that challenge detection algorithms.

The NEU-DET dataset, developed by Northeastern University of China, presents challenging conditions characterized by significant noise, uneven illumination, diverse defect morphologies, and intra-class variability, making it particularly suitable for validating robust detection algorithms. The dataset contains 1,800 images across six surface defect categories, with 300 samples per class at 200×200 pixel resolution, as depicted in Fig 6. The defect categories comprise cracking (Cr), pitting surface (Pi), rolled oxide (Ro), scratch (Sc), inclusion (In), and patch (Pa). As illustrated in the representative samples of Fig 6, each defect category exhibits distinct morphological characteristics with irregular spatial distributions and varying visual complexity. Notably, cracking samples demonstrate substantial background noise and non-uniform illumination that can obscure critical defect boundaries, potentially leading to missed detections and compromised feature learning, ultimately challenging model robustness and detection accuracy.

#### GC10-DET dataset.

**Fig 7:**
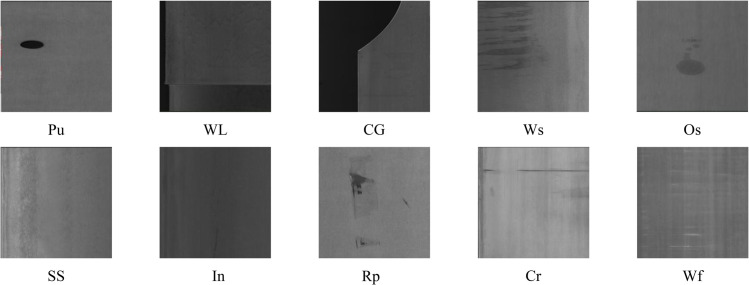
Defect category distribution in GC10-DET dataset. The ten defect classes represent diverse steel surface anomalies with varying scales, textures, and morphological characteristics.

The GC10-DET dataset comprises 2,294 high-resolution images with dimensions of 2048×1000 pixels, encompassing ten distinct steel surface defect categories as illustrated in Fig 7. The defect taxonomy includes: punching hole (Pu), weld line (Wl), crescent gap (Cg), water spots (Ws), oil spots (Os), silk spot (Ss), inclusion (In), rolled pit (Rp), crease (Cr), and waist folding (Wf). This dataset provides enhanced complexity through higher resolution imagery and increased defect category diversity, enabling comprehensive evaluation of model generalization capabilities across varied industrial surface conditions.

### Evaluation metrics

Following standard object detection evaluation protocols, we employ precision (P), recall (R), average precision (AP), mean average precision (mAP), computational complexity (GFLOPs), and model parameters (Params) as primary performance indicators. The evaluation metrics are defined as:

$$P=\frac{TP}{TP+FP}$$ (9)

$$R=\frac{TP}{TP+FN}$$ (10)

$$AP=\int_{0}^{1}P(R)\;dR$$ (11)

$$mAP=\frac{1}{n}\sum_{i=1}^{n}AP_{i}$$ (12)

where TP represents true positive detections, FP denotes false positive predictions, FN indicates false negative instances (missed detections), and *n* corresponds to the total number of defect categories. Average precision (AP) is computed as the area under the precision-recall curve for each class, while mean average precision (mAP) represents the average AP across all categories. For single-class scenarios, mAP reduces to AP.

### Experimental configuration

Experiments are conducted on a Windows-based workstation equipped with an NVIDIA RTX 3060 GPU, utilizing CUDA 12.3, PyTorch 1.12.0 framework, and Python 3.8.5 environment. Training employs a primary 9:1 training-testing partition, with selected experiments utilizing an 8:2 split for robustness evaluation. Training hyperparameters include 350 epochs, initial learning rate of 0.01, batch size of 16, momentum coefficient of 0.937, and weight decay of 0.0005, optimized through stochastic gradient descent (SGD).

### Loss function comparative analysis

Loss function design fundamentally influences model optimization by quantifying prediction-target discrepancies, with effective formulations enhancing robustness through balanced training efficiency and generalization capability. We evaluate our proposed β-FEIoU loss against established IoU variants—CIoU, DIoU, WIoU, SIoU, and EIoU—across multiple data partitioning schemes to assess performance consistency and robustness. Tables 1 and 2 present comprehensive comparative results demonstrating the superior balanced performance of β-FEIoU across all defect categories, regardless of data partitioning strategy. Under the 9:1 configuration, β-FEIoU achieves 76.0% mAP, surpassing the second-best approach by 0.3 percentage points. The more challenging 8:2 split further validates our method’s efficacy, with β-FEIoU maintaining optimal performance at 73.6% mAP compared to SIoU’s 72.6%. This consistency across varying training data availability demonstrates the robustness of our loss formulation.

**Table 1:** mAP values under different losses with a training-to-testing ratio of 9:1.

Loss	mAP50%	mAP50%					
		Cr	In	Pa	Pi	Ro	Sc
CIoU	73.0	40.1	81.5	90.7	74.7	65.0	86.0
DIoU	75.7	**48.4**	83.0	93.9	75.9	64.2	**88.9**
WIoU	72.7	40.4	76.2	90.9	75.8	68.1	84.8
SIoU	75.6	35.5	**89.0**	90.9	76.3	**78.4**	83.8
EIoU	75.1	34.2	82.2	**96.0**	**78.8**	71.8	87.4
β-FEIoU	**76.0**	44.4	86.1	95.3	77.1	70.9	82.1

**Table 2:** mAP values under different losses with a training-to-testing ratio of 8:2.

Loss	mAP50%	mAP50%					
		Cr	In	Pa	Pi	Ro	Sc
CIoU	71.1	48.2	80.7	92.4	**82.5**	49.1	74.0
DIoU	72.4	**50.6**	80.9	92.9	79.8	56.6	73.6
WIoU	72.0	42.9	**85.3**	**94.2**	77.4	52.6	79.6
SIoU	72.6	47.4	83.4	91.4	81.2	52.1	**80.4**
EIoU	72.1	47.4	82.3	92.0	81.2	56.4	73.4
β-FEIoU	**73.6**	50.3	82.2	91.1	**82.5**	**59.2**	75.6

A category-specific analysis reveals distinct performance characteristics across various loss functions. Specifically, while DIoU excels in the detection of cracking defects and EIoU optimizes patch detection, β-FEIoU demonstrates exceptional stability and balanced performance across *all* defect categories. Within the particularly challenging cracking category, our method achieves performance of 44.4% and 50.3% under different data partitioning schemes. These figures represent substantial improvements of 8.9% and 2.9% over the weakest-performing baselines, respectively. Crucially, the performance differential observed between the 9:1 and 8:2 data partitions offers critical insights into the inherent stability of different loss functions. Remarkably, β-FEIoU exhibits exceptional resilience, showing only a 2.4% performance degradation even with a significant reduction in training data. This strongly suggests superior generalization capabilities. Conversely, conventional approaches like CIoU and EIoU suffer significantly more substantial performance deterioration, indicating a heightened susceptibility to overfitting when confronted with limited training samples.

The effectiveness of β-FEIoU stems from the synergistic integration of EIoU regression loss with Focal loss classification, enhanced by our adaptive β weighting mechanism. The Focal loss component effectively addresses class imbalance between background and defect regions, while the β parameter enables dynamic adjustment based on detection difficulty and inter-class relationships. This dual mechanism accelerates convergence while enhancing discriminative capacity for subtle defect variations. Through comprehensive evaluation across diverse defect categories and data partitioning scenarios, β-FEIoU consistently demonstrates superior balanced performance and exceptional robustness for industrial surface defect detection applications.

### The impact of LSKA kernel size

LSKA employs a sophisticated attention mechanism that decomposes traditional two-dimensional convolution kernels into cascaded one-dimensional operations along horizontal and vertical axes within deep convolutional layers. We evaluate six distinct kernel dimensions: 7, 11, 23, 35, 41, and 53 pixels. Given the morphological diversity of surface defects across both datasets, which encompass substantial scale variations and heterogeneous geometric patterns including elongated, triangular, and semi-circular configurations, kernel size selection critically influences detection performance.

Tables 3 and 4 demonstrate that smaller convolution kernels consistently produce suboptimal overall accuracy due to their inherent spatial locality constraints, which limit comprehensive defect information capture across extended spatial contexts. Medium-sized kernels (11 and 23) demonstrate superior performance for fine-grained defect feature detection, particularly excelling at identifying irregular elongated or curved defect characteristics that require balanced local-global context integration. Conversely, larger kernels provide enhanced global contextual information and achieve superior accuracy for substantial defect features, demonstrating optimal performance on expansive regions with regular geometric patterns such as large patches or crescent-shaped anomalies. However, this expanded receptive field compromises spatial localization precision, thereby reducing detection efficacy for minute defect features requiring fine-grained spatial discrimination.

**Table 3:** Effects of different LSKA convolution kernel in NEU-DET Dataset.

LSKA kernel	mAP50%	mAP50%					
		**Cr**	**In**	**Pa**	**Pi**	**Ro**	**Sc**
7	75.4	51.3	84.5	**98.4**	**78.6**	64.8	82.9
11	75.5	45.5	84.0	94.8	76.1	67.1	**85.5**
23	**77.0**	**54.5**	87.1	95.9	69.9	**72.2**	82.5
35	75.2	42.9	**88.0**	92.1	76.3	69.1	83.1
41	75.4	47.0	86.5	90.9	76.1	68.3	83.7
53	75.0	49.7	86.9	92.8	75.7	63.9	81.4

**Table 4:** Effects of different LSKA convolution kernel in GC10-DET Dataset.

LSKA kernel	mAP50%	mAP50%									
		Pu	WL	CG	Ws	Os	SS	In	Rp	Cr	Wf
7	66.8	92.0	84.6	96.3	81.2	53.3	60.4	25.2	34.6	51.8	**88.4**
11	**67.2**	**94.4**	**89.8**	92.4	80.9	**68.8**	72.6	**45.5**	5.6	43.7	78.2
23	67.1	93.2	87.0	94.7	**82.7**	63.1	71.6	38.9	17.0	43.3	79.4
35	66.7	94.2	85.4	**97.5**	78.8	62.7	**74.0**	33.9	15.9	47.6	77.1
41	66.3	92.6	75.7	95.5	74.4	63.9	61.4	23.1	**39.6**	49.1	87.7
53	66.6	92.8	81.6	95.7	77.6	67.2	55.3	25.4	30.4	**55.6**	83.9

The empirical results reveal an optimal trade-off at kernel size 23 for NEU-DET (77.0% mAP) and kernel size 11 for GC10-DET (67.2% mAP), where sufficient contextual information is captured without sacrificing the spatial precision essential for detecting subtle surface anomalies. This dataset-dependent optimal kernel size reflects the distinct defect characteristics and complexity levels inherent in each industrial surface inspection scenario.

### Ablation study

To validate the effectiveness of our proposed architectural enhancements, we conduct comprehensive ablation experiments using YOLOv8n as the baseline framework. Table 5 presents the ablation results for each module configuration on the NEU-DET dataset. The ablation results demonstrate distinct performance characteristics for each architectural component. Initially, while the WASPP module (YOLOv8n_1) introduces substantial parameter overhead and computational complexity, it achieves notable detection improvements, yielding 76.5% mAP compared to the 73.0% baseline. The SAConv module in isolation (YOLOv8n_2) exhibits minimal performance enhancement, achieving only marginal improvement with negligible parameter increase. This limited effectiveness suggests that SAConv requires synergistic integration with contextual attention mechanisms to realize its full potential. The LSKA attention mechanism (YOLOv8n_3) demonstrates substantial standalone effectiveness, achieving 75.2% mAP with minimal computational overhead. This 2.2% improvement validates LSKA’s capacity to enhance contextual feature relationships and direct model attention toward critical defect characteristics. The β-FEIoU loss function (YOLOv8n_4) significantly improves regression performance, achieving 76.0% mAP while maintaining identical parameter count and computational complexity as the baseline, confirming the effectiveness of our adaptive loss formulation. Progressive module integration reveals compelling synergistic effects. Combining β-FEIoU with LSKA (YOLOv8n_5) yields 77.0% mAP, demonstrating that enhanced loss optimization facilitates improved attention mechanism performance. The addition of SAConv (YOLOv8n_6) produces substantial improvement to 79.2% mAP, validating our hypothesis that SAConv effectiveness depends on adequate contextual interaction provided by LSKA. This configuration enables SAConv to effectively extract shallow features, enrich spatial information, and emphasize multi-scale receptive field importance.

**Table 5:** The ablation results of each module.

Model	Loss	LSKA	SAConv	WASPP	mAP50%	Parame	GFLOPs
Yolov8n	—	—	—	—	73.0	**2.87**	**8.1**
Yolov8n_1	—	—	—	√	76.5	5.45	12.3
Yolov8n_2	—	—	√	—	73.2	2.88	8.5
Yolov8n_3	—	√	—	—	75.2	3.04	8.4
Yolov8n_4	√	—	—	—	76.0	**2.87**	**8.1**
Yolov8n_5	√	√	—	—	77.0	2.96	8.3
Yolov8n_6	√	√	√	—	79.2	3.05	8.7
ours	√	√	√	√	**80.4**	5.64	12.8

**Fig 8:**
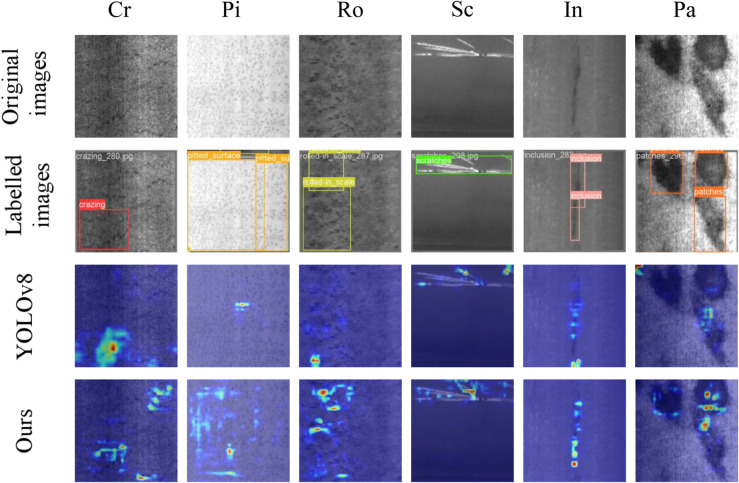
Comparative feature extraction visualization through HiResCam analysis. Heat maps demonstrate superior defect localization capabilities of our proposed architecture compared to baseline YOLOv8n across representative defect categories, revealing enhanced sensitivity to subtle anomalies and improved spatial feature extraction.

The complete architecture incorporating all proposed components achieves 80.4% mAP, representing a remarkable 7.4% improvement over the baseline YOLOv8n. While this configuration incurs increased computational cost, the substantial performance gains justify the computational overhead for industrial defect detection applications requiring high accuracy. The systematic ablation study validates each component’s contribution and demonstrates the synergistic benefits of integrated architectural enhancements. To comprehensively assess feature extraction capabilities, we employ HiResCam visualization comparing our enhanced architecture with the baseline implementation, as illustrated in Fig 8. The comparative analysis reveals superior feature extraction performance across diverse defect categories. For cracking defects, while only single defects receive explicit annotations, multiple unmarked anomalies exist within images. Our model successfully identifies several unlabeled instances, demonstrating enhanced sensitivity to subtle defect patterns. Similarly, for pitting defects characterized by extensive spatial distribution, the baseline model exhibits inadequate feature learning, whereas our architecture demonstrates comprehensive defect extraction capabilities. Consistent improvements across all defect categories validate our model’s enhanced feature extraction efficacy and substantiate the architectural enhancements’ effectiveness.

### Comparative performance analysis

To comprehensively validate our proposed architecture’s effectiveness, we conduct extensive comparisons against state-of-the-art detection frameworks and contemporary approaches. We standardize input image dimensions across all evaluated models to ensure consistent experimental conditions. Open-source architectures (YOLOv5, YOLOv8, YOLOv11, YOLOX, Faster R-CNN, SSD, RT-DETR) undergo retraining on the NEU-DET dataset using their respective default hyperparameter configurations and identical training protocols. For contemporary methods referenced in recent literature [39–42], we implement their approaches based on published methodological descriptions and apply consistent experimental conditions matching our proposed framework. Unspecified parameters align with our implementation standards to maintain fair comparative evaluation.

Table 6 demonstrates that our proposed architecture achieves superior overall detection performance, attaining 80.4% mAP while maintaining reasonable computational efficiency. Although our model exhibits higher parameter count than certain lightweight architectures, it delivers the highest detection accuracy across all evaluated methods. The performance analysis reveals several key insights regarding architectural trade-offs and detection capabilities. Examining category-specific performance, our approach demonstrates superior or comparable accuracy across most defect types. Notably, our model achieves exceptional performance on inclusion and pitting defects, significantly outperforming all comparative methods. For patch defects, our approach achieves 95.1% mAP, matching the performance of leading methods while maintaining superior overall balance. The only category where our method shows marginal underperformance is cracking defects, where Wu et al. [40] and YOLOv11n achieve 53.5% and 51.5% respectively, compared to our 49.4%. This limitation likely stems from the inherently challenging nature of crack detection, characterized by subtle, elongated features that require specialized architectural considerations.

**Table 6:** Performance comparison with state-of-the-art detection methods on NEU-DET dataset.

Model		mAP50%					mAP50%	Param(M)	GFLOPs
	Cr	In	Pa	Pi	Ro	Sc			
Yolov5n [43]	37.0	79.2	94.5	76.7	66.0	76.9	71.7	1.69	4.2
Yolov5s [43]	48.7	84.4	95.0	72.7	61.6	76.6	73.2	6.71	16.0
Yolov8n [44]	40.1	81.5	90.7	74.7	65.0	86.0	73.0	2.87	8.1
YoloX [45]	37.4	80.9	91.5	77.2	49.0	87.7	70.6	8.94	26.8
Yolov11n [44]	51.5	90.2	94.7	74.8	72.3	84.5	78.0	2.47	6.3
SSD [9]	39.8	70.1	79.2	80.1	54.9	86.2	68.4	24.8	61.7
Faster-RCNN [6]	43.5	74.0	86.5	80.4	78.7	74.3	72.9	136.9	396.9
RT-DETR [13]	48.6	89.7	88.8	65.6	65.2	88.4	74.4	18.9	57.0
Dong et al. [39]	45.3	87.7	95.2	79.6	66.7	83.7	76.4	2.30	6.4
Wu et al. [40]	53.5	85.4	95.3	77.5	70.3	86.7	78.1	**1.60**	**3.4**
Lu et al. [41]	46.0	84.7	95.8	73.9	69.7	78.4	74.8	2.45	5.0
You et al. [42]	46.8	80.5	95.2	72.6	66.5	81.3	73.8	3.48	8.1
Ours	49.4	90.6	95.1	83.2	76.2	87.8	**80.4**	5.64	12.8

Computational efficiency analysis reveals favorable trade-offs compared to resource-intensive architectures. Relative to Faster R-CNN, our model demonstrates substantially reduced computational requirements while achieving superior detection accuracy. Similarly, compared to SSD and RT-DETR, our architecture provides enhanced accuracy with significantly lower parameter overhead and computational complexity. While lightweight approaches such as Wu et al. [40] achieve superior parameter efficiency, our method substantially surpasses their detection performance by 2.3 percentage points, justifying the moderate computational overhead. Our approach demonstrates particularly strong performance on challenging defect categories requiring fine-grained feature discrimination, establishing its effectiveness for industrial surface inspection applications where detection accuracy is paramount.

### Generalization experiment

To evaluate the generalization capability of our proposed architecture, we conduct performance assessment on the GC10-DET dataset as an independent validation benchmark. Table 7 presents the generalization results, where our method demonstrates superior performance across multiple defect categories compared to state-of-the-art detectors. Particularly noteworthy is the exceptional accuracy of 99.4% achieved for punching defects, establishing new performance benchmarks in this category.

**Table 7:** Generalization performance comparison across different detection architectures.

Model	mAP50%										mAP50%
	Pu	WL	CG	Ws	Os	SS	In	Rp	Cr	Wf	
YOLOv5n [43]	99.0	88.8	98.1	83.5	63.2	59.0	50.8	12.3	34.8	99.5	68.9
YOLOv5s [43]	98.9	91.5	93.4	82.2	73.6	71.3	44.4	31.8	53.7	72.7	71.3
YOLOv8n [44]	97.8	91.3	96.7	85.4	71.1	75.5	40.1	6.2	45.9	78.1	68.8
YOLOv11n [45]	99.3	78.5	97.5	79.7	69.8	79.8	34.3	21.9	37.2	94.1	69.2
Faster-RCNN [6]	83.1	85.3	75.0	60.1	44.6	41.5	24.4	1.5	50.5	70.3	53.6
SSD [9]	86.5	81.2	88.8	69.5	38.9	49.7	0.4	0.6	19.8	79.9	51.4
RT-DETR [13]	89.9	92.7	96.6	73.0	69.1	57.5	29.5	31.8	51.7	95.1	68.7
Dong et al. [39]	98.8	93.7	96.2	83.8	74.1	74.3	39.7	2.4	51.0	78.4	69.2
Wu et al. [40]	98.3	91.1	96.8	88.7	65.5	76.1	29.8	19.3	50.1	83.6	69.9
Lu et al. [41]	93.9	88.3	96.4	83.4	67.3	76.2	47.5	3.8	46.0	76.0	67.9
You et al. [42]	99.1	91.9	96.9	80.7	73.1	71.1	27.7	3.0	56.0	78.6	67.8
Ours	99.4	94.4	96.8	84.7	76.0	73.6	44.0	16.0	59.0	77.4	**72.1**

Nevertheless, performance degradation occurs for the indentation defects, primarily attributed to severe class imbalance inherent in the dataset. Specifically, the substantial disparity between 85 indentation samples and 884 filament samples creates a pronounced imbalance that impedes effective feature distribution learning during training. This imbalance exacerbates domain shift complications [46] and exacerbates the learning difficulty for under-represented classes. Consequently, the model exhibits inherent bias toward high-frequency classes such as filaments, leading to systematic misclassification of indentation defects. Despite these challenges, our architecture achieves 72.1% overall detection accuracy, surpassing existing leading detectors by a substantial margin of 3.3% over YOLOv8n. This performance gain validates the robust generalization capability across diverse datasets and demonstrates considerable potential for broader industrial deployment scenarios.

### Qualitative analysis

**Fig 9:**
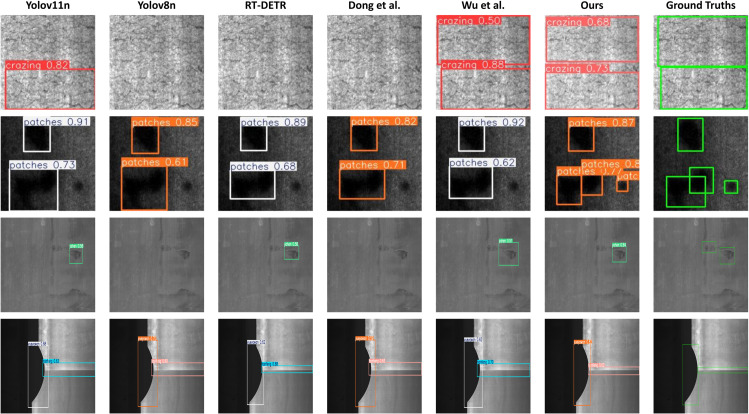
Comparative visualization of detection performance across different methods on industrial defect samples.

Fig 9 presents a comprehensive qualitative comparison, demonstrating the superior detection performance of the proposed EFEN-YOLOv8 architecture across diverse industrial defect scenarios. The visualization results reveal that while competitive methods including YOLOv11n, YOLOv8n, RT-DETR, and recent approaches by Dong et al. and Wu et al. exhibit varying performance across different defect categories, our proposed method consistently delivers robust detection with high confidence scores.

Notably, for challenging defect categories such as surface cracking, our method achieves significantly higher confidence scores compared to baseline approaches while maintaining exceptional localization accuracy through precisely fitted bounding boxes. The patch detection results demonstrate particularly strong performance, successfully identifying and labeling each defect independently, thereby validating our method’s capability to effectively distinguish negative samples. This multi-category defect analysis validates the contribution of our architectural improvements toward enhanced detection robustness and accuracy. Although certain competitive methods occasionally achieve higher confidence scores in isolated cases, they demonstrate inconsistent performance across the comprehensive evaluation spectrum, frequently producing loose bounding boxes or failing to detect subtle defects entirely. Visualization confirms that our results establish the practical feasibility of the proposed architecture in real-world industrial testing applications.

### Robustness analysis using random data splits

#### Robustness analysis for ablation experiments.

To rigorously assess the efficacy of each proposed module, comprehensive statistical significance testing was conducted. This involved five independent experiments, each initialized with distinct random seeds, performed on the NEU-DET dataset using a 9:1 train-test split. Detailed statistical analysis, presented in Table 8, encompasses mean performance, standard deviation, 95% confidence intervals, and paired t-test results comparing each module’s contribution against the baseline YOLOv8n.

**Table 8:** Statistical significance analysis of ablation components across 5 random splits on NEU-DET dataset. The evaluation indicators mainly include the mean (μ), standard deviation (σ), 95% confidence interval, improvement over baseline (Δ), and p-value of mAP scores.

Module	Run 1	Run 2	Run 3	Run 4	Run 5	μ±σ	95% CI	Δ	*p* *-value*
Baseline	73.0	72.5	72.6	73.1	73.0	72.8±0.27	[72.49, 73.19]	–	–
LSKA	75.2	75.4	75.9	75.4	75.8	75.5±0.28	[75.20, 75.88]	+2.7	<0.001
SAConv	73.2	73.5	74.2	73.8	73.3	73.6±0.41	[73.08, 74.12]	+0.8	<0.05
β-FEIoU	76.0	74.9	76.0	75.5	76.2	75.7±0.53	[75.06, 76.38]	+2.9	<0.001
WASPP	76.5	75.9	76.2	76.6	76.1	76.3±0.28	[75.92, 76.60]	+3.5	<0.001

The statistical analysis unequivocally demonstrates that all proposed modules yield significant performance improvements, bolstering confidence in their individual efficacy. Specifically, WASPP registers the most substantial individual gain, improving performance by +3.5% (p < 0.001). This is closely followed by β-FEIoU, which contributes +2.9% (p < 0.001), and LSKA, yielding a +2.7% enhancement (p < 0.001). Though SAConv exhibits the most modest standalone improvement, exhibiting a +0.8% gain (p < 0.05), its contribution nonetheless remains statistically significant. Crucially, the relatively modest performance of SAConv in isolation validates a key architectural hypothesis: that this module’s full potential is unlocked only through synergistic integration with contextual attention mechanisms, particularly LSKA. This synergy is powerfully evidenced by the substantial performance gains observed in combined configurations (Table 5). Further affirming the robustness and reproducibility of our experimental results are the uniformly low standard deviations (σ≤0.53) and narrow confidence intervals observed across all configurations. Collectively, these findings offer compelling statistical evidence that each proposed component verifiably and significantly contributes to the final architecture’s superior detection performance.

#### Robustness analysis of model performance.

To ensure experimental robustness and statistical reliability, we employed a rigorous validation framework. Specifically, five independent experiments, each initialized with a distinct random seed, were conducted on both NEU-DET and GC10-DET datasets, evaluated across two train/test ratios (8:2 and 9:1). Crucially, dataset shuffling was performed in each iteration to ensure statistical independence between training and testing partitions. Table 9 presents the detailed results of this five-fold experimental validation, including mean (μ), standard deviation (σ), and 95% confidence intervals for mAP scores. Our statistical analysis highlights significant performance disparities across varying configurations and datasets. Notably, increasing the training data proportion from 80% to 90% consistently yielded substantial improvements across both datasets: NEU-DET registered a 4.3 percentage point enhancement (from 76.1% to 80.4%), while GC10-DET exhibited an even more pronounced 11.3 percentage point improvement (from 60.8% to 72.1%). These gains unequivocally demonstrate that our proposed architecture significantly benefits from larger training sets, particularly for challenging datasets characterized by intricate defect patterns.

**Table 9:** Statistical analysis of experimental results across 5 random splits. The evaluation indicators mainly include the mean (μ), standard deviation (σ), and 95% confidence interval of mAP scores.

Dataset	Split	Run 1	Run 2	Run 3	Run 4	Run 5	μ±σ	95% CI
NEU-DET	8:2	76.6	76.1	75.9	75.8	76.3	76.1±0.31	[75.72, 76.48]
NEU-DET	9:1	79.9	80.8	80.2	80.7	80.4	80.4±0.36	[79.95, 80.85]
GC10-DET	8:2	64.3	59.4	62.2	58.4	59.3	60.8±2.51	[57.48, 64.12]
GC10-DET	9:1	71.0	73.3	69.8	72.9	73.5	72.1±1.63	[70.09, 74.11]

Underscoring fundamental differences in dataset complexity is the consistently superior performance observed on NEU-DET relative to GC10-DET. NEU-DET, for instance, features inherently more distinguishable defect patterns, thereby facilitating more effective model learning. This disparity is likely attributable to severe class imbalance within GC10-DET, where certain defect categories possess insufficient training samples. Such an imbalance impedes adequate feature learning, inevitably resulting in degraded performance for these underrepresented classes. Furthermore, variance analysis yields critical insights into model stability and dataset characteristics. NEU-DET demonstrates exceptionally low standard deviations (σ≤0.36) across all experimental configurations, signifying robust and consistent performance irrespective of stochastic initialization. Conversely, GC10-DET exhibits substantially elevated variance (σ=1.63–2.51), most pronounced in the 8:2 partitioning scheme, indicating heightened susceptibility to training instabilities and convergence difficulties. The constrained confidence intervals observed for NEU-DET ([75.72,76.48] and [79.95,80.85]) attest to superior precision in performance estimation, whereas the substantially broader intervals characterizing GC10-DET ([57.48,64.12] and [70.09,74.11]) underscore the intrinsic complexity and detection variability inherent within this challenging dataset.

#### Confusion matrix analysis and performance insights.

**Fig 10:**
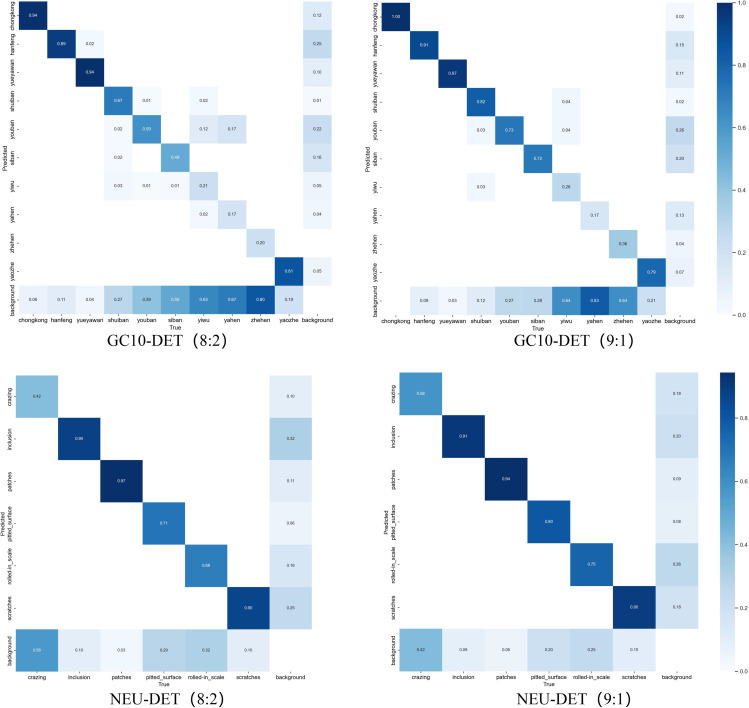
Confusion matrices demonstrating classification performance under different training-testing data splits.

Fig 10 presents confusion matrices under different training-testing ratios, providing comprehensive insights into the model’s classification capabilities and performance characteristics across defect categories. On the GC10-DET dataset, the proposed EFEN-YOLOv8 demonstrates exceptional classification performance, with most categories achieving diagonal values exceeding 0.70. Particularly noteworthy are the hanfeng, yueyawan, and shuiban defects, which exhibit superior detection accuracy. This high-precision performance indicates effective feature extraction and discrimination capabilities inherent in the enhanced architecture. Similarly, on the NEU-DET dataset, categories including inclusion, patches, and scratches maintain robust detection accuracy above 0.90, validating the model’s effectiveness across diverse industrial scenarios.

The analysis reveals distinctive performance patterns that underscore the complexity inherent in real-world defect detection challenges. Categories exhibiting similar visual characteristics, such as crazing patterns that closely resemble natural surface variations, present intrinsic classification complexity—a reflection of the challenging nature of industrial quality control rather than architectural deficiencies. The comparative performance across different train-test ratios (9:1 and 8:2) demonstrates the model’s adaptability to varying data distributions while maintaining consistent improvements over baseline methods across all configurations.

Despite achieving state-of-the-art performance, certain challenging scenarios warrant continued investigation. These include the detection of highly subtle defects existing at the classification boundary between normal and defective samples, and optimization strategies for severely imbalanced datasets where specific defect types occur infrequently in industrial environments. In particular, the imbalance or scarcity of defective samples has resulted in poor detection performance for certain types. Nevertheless, these observations provide clear directions for future research while affirming the significant advancement achieved by the proposed architecture in enhancing practical defect detection capabilities.

## Conclusion

This paper presents EFEN-YOLOv8, a novel defect detection architecture incorporating four key innovations. The SAConv module enhances shallow-layer feature localization through weighted attention mechanisms, enabling early-stage defect identification with differential feature emphasis. The proposed β-FEIoU loss function addresses class discrimination challenges while mitigating positive-negative sample imbalance, simultaneously accelerating convergence and enhancing regression precision. The integrated LSKA attention mechanism amplifies defect feature focus following sample balance optimization. The WASPP module facilitates multi-scale feature fusion with adaptive weighting to emphasize critical feature importance.

Comprehensive experimental validation demonstrates the architecture’s effectiveness across multiple evaluation scenarios. On NEU-DET, our model achieves 80.4% detection accuracy without preprocessing under 9:1 data partitioning, with robust performance of 76.1% under the more challenging 8:2 split, demonstrating substantial improvements across all defect categories. Notably, the challenging Cr defect category exhibits remarkable enhancement from 37.0% to 49.4% mAP, while maintaining superior performance for remaining defect types. Statistical significance analysis through five-fold cross-validation with different random seeds confirms the model’s reliability, yielding exceptionally low variance (σ≤0.36) and narrow confidence intervals, indicating consistent performance across diverse experimental configurations. On GC10-DET, the model attains 72.1% precision, representing a 3.3% improvement that validates the architecture’s robustness and generalization capabilities across heterogeneous datasets. While achieving significant improvements over existing approaches, this work identifies areas where continued research can further advance surface defect detection capabilities. The confusion matrix analysis reveals that certain challenging scenarios, including highly subtle defects near classification boundaries and severely imbalanced industrial datasets, represent ongoing research opportunities rather than fundamental limitations. The computational efficiency positions the model favorably for industrial deployment, though optimization opportunities exist for resource-constrained environments. The performance variations across different datasets demonstrate the inherent complexity of diverse industrial applications while maintaining substantial improvements over baseline methods, indicating promising directions for domain adaptation techniques.

Future research directions include algorithmic refinement through dataset optimization and architectural enhancement to minimize false detection rates while improving overall accuracy and robustness. Proposed methodological improvements include noise mitigation through advanced filtering techniques, CLAHE-based [47] noise suppression, sophisticated image enhancement algorithms, and data augmentation via adversarial generative networks [48]. Additionally, model pruning strategies will be investigated to reduce computational overhead, thereby enhancing inference speed and overall detection efficiency.
